# Effect of habitat degradation on competition, carrying capacity, and species assemblage stability

**DOI:** 10.1002/ece3.2977

**Published:** 2017-06-17

**Authors:** Edoardo Calizza, Maria Letizia Costantini, Giulio Careddu, Loreto Rossi

**Affiliations:** ^1^ Department of Environmental Biology Sapienza University of Rome Rome Italy; ^2^ CoNISMa Rome Italy

**Keywords:** habitat degradation, invertebrates, niche overlap, optimal foraging, population dynamics, seagrass, stable isotopes, trophic niche

## Abstract

Changes in species’ trophic niches due to habitat degradation can affect intra‐ and interspecific competition, with implications for biodiversity persistence. Difficulties of measuring species’ interactions in the field limit our comprehension of competition outcomes along disturbance gradients. Thus, information on how habitat degradation can destabilize food webs is scarce, hindering predictions regarding responses of multispecies systems to environmental changes. Seagrass ecosystems are undergoing degradation. We address effects of *Posidonia oceanica* coverage reduction on the trophic organization of a macroinvertebrate community in the Tyrrhenian Sea (Italy), hypothesizing increased trophic generalism, niche overlap among species and thus competition and decreased community stability due to degraded conditions. Census data, isotopic analysis, and Bayesian mixing models were used to quantify the trophic niches of three abundant invertebrate species, and intra‐ and interspecific isotopic and resource‐use similarity across locations differing in seagrass coverage. This allowed the computation of (1) competition strength, with respect to each other and remaining less abundant species and (2) habitat carrying capacity. To explore effects of the spatial scale on the interactions, we considered both individual locations and the entire study area (“‘meadow scale”). We observed that community stability and habitat carrying capacity decreased as *P. oceanica* coverage declined, whereas niche width, similarity of resource use and interspecific competition strength between species increased. Competition was stronger, and stability lower, at the meadow scale than at the location scale. Indirect effects of competition and the spatial compartmentalization of species interactions increased stability. Results emphasized the importance of trophic niche modifications for understanding effects of habitat loss on biodiversity persistence. Calculation of competition coefficients based on isotopic distances is a promising tool for describing competitive interactions in real communities, potentially extendible to any subset of ecological niche axes for which specimens’ positions and pairwise distances can be obtained.

## 1 Introduction

Habitat degradation is acknowledged to be a major driver of biodiversity loss and species abundance reduction (Krauss et al., 2010; SCBD 2010). However, our mechanistic understanding of how degradation of habitats affects food web stability and leads to species loss is limited, particularly concerning aquatic habitats. Competitive interactions are expected to play a central role in both population and community dynamics, underlying the stable coexistence of populations and structuring natural communities along environmental gradients (Holdridge, Cuellar‐Gempeler, & terHost, 2016; Shea & Chesson, 2002; Whittaker & Levin, 1975). Limited resource availability, often associated with degraded habitats, may enhance competition, leading to reduced equilibrium densities and the exclusion of subordinate competitors, even between flexible‐diet consumers which do not compete in undisturbed environments (Auer & Martin, 2013; Boström‐Einarsson, Bonin, Munday, & Jones, 2014). However, difficulties with the field measurement of interaction strength limit our comprehension of competition outcomes in natural communities along disturbance gradients, hindering prediction of the response of real multispecies systems to environmental changes.

Optimal foraging theories posit trophic niche broadening as a consequence of reduced per capita food availability, where consumers relying on insufficient preferred food items are forced to add less profitable resources to their diet, hence widening their trophic niche (Pyke, Pulliam, & Charnov, 1977; Rossi, di Lascio, Carlino, Calizza, & Costantini, 2015). Both model and field studies have demonstrated foraging optimization in species assemblages and recovery patterns after disturbance in food webs (Beckerman, Petchey, & Warren, 2006; Calizza, Costantini, Rossi, Carlino, & Rossi, 2012; Kondoh, 2003; Rossi et al., 2015), with less diverse and more interconnected generalist‐dominated communities associated with disturbed conditions and/or degraded habitats (Calizza et al., 2012; Calizza, Costantini, & Rossi, 2015; Munday, 2004; O'Gorman, Fitche, & Crowe, 2012; Valladares, Cagnolo, & Salvo, 2012). In addition, intraspecific competition has been shown to promote trophic generalism within populations due to differentiation in food use among conspecifics (Araújo, Langerhans, Giery, & Layman, 2014; Bolnick, 2001; Svanbäck & Bolnick, 2007). Thus, food webs in degraded habitats may be characterized by stronger competition and lower carrying capacities and equilibrium densities, along with lower stability (i.e., local Lyapunov stability) associated with higher niche overlap between species (Costantini et al., 2012; May, 1974a; Rooney, McCann, Gellner, & Moore, 2006; Whittaker & Levin, 1975).

Among marine environments, seagrass habitats are biodiverse and productive ecosystems experiencing decline due to global changes including temperature rise, species invasion, and anthropogenic disturbance of coastal areas (Hemminga & Duarte, 2000; Orth et al., 2006). Declining biodiversity and secondary productivity have been reported in association with the declining habitat complexity and resource availability that accompanies reductions in seagrass coverage (Calizza, Costantini, et al., 2013; Hemminga & Duarte, 2000; Orth et al., 2006). Nevertheless, the ecological mechanisms underlying this relationship are poorly understood. Understanding when and how habitat degradation affects biodiversity organization by modifying resources at the base of the food web, species’ trophic niches, and the strength of interspecific interactions would shed light on the mechanisms by which climate‐ and human‐induced habitat changes affect biodiversity persistence in these high‐value ecosystems. By analyzing C and N stable isotopes as tracers of the contribution of resources to consumer diets (Bašić & Britton, 2016; Bentivoglio et al., 2015; Careddu et al., 2015; Rossi et al., 2015; Yao, Huang, Xie, & Xu, 2016), this study addresses the effect of *Posidonia oceanica* (L.) Delile habitat degradation on competition for food sources, carrying capacity, and the stability of a benthic invertebrate community associated with *P. oceanica* litter. Stable isotopes have been shown to be useful for studying nutrient inputs in coastal areas and the diet of fauna associated with *P. oceanica* (Calizza, Costantini, et al., 2013; Jona‐Lasinio et al., 2015; Orlandi et al., 2014; Rossi, Costantini, Carlino, di Lascio, & Rossi, 2010; Vizzini, Sarà, Michener, & Mazzola, 2002), and C and N isotopic data have provided quantitative descriptions of species’ trophic niche width and overlap in marine environments (Jackson, Inger, Parnell, & Bearhop, 2011; Layman, Qattrocchi, Peyer, & Allgeier, 2007; Swanson et al., 2015).

Here, we focused on the variation in isotopic niche and resource use by the three most abundant species in the invertebrate community and included the effects of competition with the remaining less abundant species. Based on census data for populations and pairwise isotopic distance between both conspecific and nonconspecific organisms, we propose a method to obtain a measure of competition strength based on the comparison of intra‐ and interspecific isotopic population similarity. Bayesian isotopic mixing models were also applied, in order to provide a second measure of interaction strength based on the proportional contribution of resources to the species’ diets in accordance with Levins (1968). This made it possible to account for uncertainty in isotopic signatures and isotopic fractionation occurring between trophic levels (Parnell et al., 2013) and to compare isotopic‐ and diet‐based competition coefficients. In order to (1) describe the effects of habitat degradation on competition strength and species assemblage stability and (2) avoid the potential confounding effects of spatial variations in the isotopic composition of resources (Araújo, Bolnick, Machado, Giaretta, & dos Reis, 2007; Flaherty & Ben‐David, 2010), interspecific interaction strengths were quantified at the local scale (Calizza et al., 2012; Careddu et al., 2015; Rossi et al., 2015) and the meadow scale (i.e., the three location‐scale communities considered as a whole). This, in association with census data, made it possible to describe changes in the carrying capacity for each population, to forecast the outcomes of competition between species pairs, and to quantify the local stability of the species assemblage along a meadow degradation gradient on multiple spatial scales. Specifically, we hypothesized that seagrass habitat degradation and the associated depletion of resources are associated with greater trophic niche widths among the invertebrate populations and consequently: (1) lower intraspecific trophic similarity, (2) greater interspecific competition strength, and (3) lower community stability. In addition, we considered the indirect effects of competition (Basset & Rossi, 1990; Lawlor, 1979) and the spatial scale of interactions (Basset, 1995; Durrett & Levin, 1998) as factors potentially explaining stability mechanisms in the *P. oceanica*‐dwelling community.

## 2 Materials and Methods

### 2.1 Study area

The study area was located near the central Tyrrhenian coast of Italy, within an area where *P. oceanica* meadows extend discontinuously for 40 km along the coastline. In this area, the conservation status of the *P. oceanica* meadows is lower at the north end (Paticchio, 2013). Intense illegal trawling during the past century increased coastal urbanization and water turbidity due to the presence of stream mouths are considered the main causes of meadow degradation (Paticchio, 2013). Water turbidity can increase during autumn‐winter, when rainfall peaks. Within this area, we selected the meadow characterized by the highest conservation status, just off the “Salt marsh Nature Reserve” of Tarquinia (Vt) (42°12′N 11°42′E) (Paticchio, 2013). The presence of the Reserve has protected this area from intense urbanization and the intensification of controls since the 1990s has reduced illegal fishing in the area. Although the current general conditions do not differ substantially from those of the 1990s, the meadow is characterized by varying degrees of coverage.

Samplings were carried out at a fixed depth of 6 m in three locations differing in the degree of *P. oceanica* coverage. Coverage was estimated in circular areas of 60 m in diameter (2,826 m^2^ surface area) by two scuba divers and by photographic analysis. Each diver operated independently during a preliminary survey, providing two independent visual estimates per location. The degree of coverage varied between locations but was highly homogeneous within each location. Accordingly, we defined a high‐coverage location (“H,” coverage: 92.5 ± 2.5%), an intermediate‐coverage location (“I,” coverage: 70.0 ± 5.0%), and a low‐coverage location (“L,” coverage: 50.0 ± 5.0%) (Kruskal–Wallis test and Mann–Whitney pairwise comparisons: Hc = 9.85, *p* < .01). Locations H and L were 1,850 m apart, with location I being positioned half‐way between the two. Macroinvertebrates were sampled using litterbags (2‐cm mesh size) anchored to the meadow bed, each containing 20‐g dry weight of *P. oceanica* leaf litter (Costantini, Rossi, Fazi, & Rossi, 2009). Two sampling sites per location were chosen. Six litterbags were placed randomly at each sampling site (making a total of 12 litterbags per sampling location), as far as possible from the edge of the seagrass patch in order to represent environmental conditions characterizing *P. oceanica* patches across three levels of seagrass coverage. The minimum distance between litterbags within each site was 10 m. Sediments, attached *P. oceanica* leaves (both with and without evident epiphyte colonization) and leaf litter, representing the trophic sources at the base of the macroinvertebrate food web, were harvested together with macroinvertebrates at each location (Table S1). This study relies on census and isotopic data presented in Calizza, Costantini, et al. (2013), where a detailed description of invertebrate and resource samplings, processing and species’ isotopic data can be found. Specifically, a total of 39 benthic macroinvertebrate species were found and the community composition differed between locations. Macroinvertebrate density decreased with meadow degradation (one‐way ANOVA and Tukey's pairwise comparisons, *p* < .01), that is, from location H (34.4 ± 6.0 individuals per litterbag) to location L (16 ± 5.2 individuals per litterbag), and species richness was higher in H (26 species) and lower in I and L (both 19 species). In addition, the percentage of both coarse (>1 mm) and ultrafine (<0.56 mm) sediments accounted for by organic matter decreased from H to L. The Percent Ash Free Dry Mass (AFDM%) of coarse sediment was 6.9 ± 0.1% in H and 3.2 ± 0.5% in L (one‐way ANOVA and Tukey's test, *p* < .05), while the AFDM% of ultrafine sediment (the richest fraction for organic matter content) was 8.1 ± 0.2% in H, with ultrafine sediment being absent from locations I and L. Both these aspects suggest that locations I and L were characterized by limiting conditions as a consequence of habitat degradation, as reported for other seagrass habitats (Bell, Brooks, Robbins, Fonseca, & Hall, 2001; Boström, Jackson, & Simenstad, 2006; Bowden, Rowden, & Attrill, 2001; Orth et al., 2006; Van der Heide et al., 2007). In the text, we will refer to “location scale” to indicate the sampling location spatial scale (i.e., accounting for differences between H, I, and L) and to “meadow scale” to indicate results describing the density and trophic organization of species in the study area as a whole (i.e., when not accounting for differences between sampling locations, see below).

### 2.2 Target species

We focused on the three most abundant species: *Microdeutopus obtusatus* Myers (Amphipoda), *Athanas nitescens* Leach (Decapoda), and *Cymodoce truncata* Leach (Isopoda) as Target Species (TS) (sensu Lawlor, 1979) undergoing competition with each other and with the remaining nontarget species (NTS) in the community. These three species were chosen as they (1) accounted for 70% of macroinvertebrate organisms in H, 84% in I and 76% in L, as well as 76% of the overall invertebrate community at the meadow scale; (2) were present at all sampling locations; (3) were characterized by similar habitat use and foraging behavior, all having the potential for active swimming if necessary, but preferring to search for shelter and remain inconspicuous, and (4) displayed similar body size (specimens considered in this study were between 11 and 14 mm in length for *M. obtusatus*, between 12 and 16 mm for *A. nitescens*, and between 11 and 15 mm for *C. truncata*), which can be an important determinant of metabolic rates and interspecific competition outcomes (Basset, 1995; Brown, Gillooly, Allen, Savage, & West, 2004; di Lascio, Rossi, & Costantini, 2011). In addition, from points (3) and (4), it may be supposed that these three species are subject to similar predatory pressure, the effect of which is not explicitly accounted for in this study (Calizza, Rossi, & Costantini, 2013; Lima, 2002; Mancinelli, Costantini, & Rossi, 2007).

### 2.3 δ^13^C and δ^15^N distribution and mixing models

For each target species at each location, population‐wide niche metrics were applied to invertebrate isotopic data in accordance with Jackson et al. (2011) and Layman, Arrington, Montaña, and Post (2007), using *stable isotope Bayesian ellipses in R* (SIBER) as part of the R statistical computing package (R Development Core Team 2012). The range (i.e., the difference between maximum and minimum values, CR) and variance (σ^2^) of δ^13^C were considered to be measures of the range of exploited resources and monodimensional niche width, respectively (Layman, Arrington, et al., 2007; Layman, Qattrocchi, et al., 2007; Rossi et al., 2015; Sanders, Vogel, & Knop, 2015). Isotopic niche space was calculated as the Standard Ellipse Area corrected for degrees of freedom (SEAc) encompassing the core (i.e., around 40%) of isotopic observations for each population, along with the isotopic total area (TA) occupied by specimens. Distribution of SEAc values obtained for each species was pairwise‐compared between locations by means of Welch's *t*‐test, which made it possible to account for unequal variance in SEAc distributions. The overlap between species pairs’ TA and SEAc at both the location and meadow scale was also calculated (SIBER analysis, Jackson et al., 2011). Overlaps between species pairs are expressed as the percentage of the TA or SEAc of each species.

At the location scale, intraspecific isotopic dissimilarity at the individual level (ND_*ii*_) was quantified as the mean isotopic (i.e., Euclidean) distance between each specimen and all remaining conspecifics within each sampling location. The mean intraspecific isotopic dissimilarity for each population (MND_*ii*_) was then obtained as the mean ND_*ii*_ value of all specimens in that population. The higher the MND_*ii*_, the higher the mean isotopic dissimilarity between conspecifics at each location. Similarly, for each TS specimen, the mean isotopic distance from each nonconspecific specimen (NDij) belonging to any other species in the community (both target and nontarget species) was also calculated, obtaining a measure of mean interspecific isotopic dissimilarity between species pairs (MNDij).

The δ^13^C and δ^15^N values of resources did not vary significantly across locations (PERMANOVA, *p* > .05; Table S1). However, the isotopic distribution of each species (both TS and NTS) at the meadow scale was obtained from the individual δ^13^C and δ^15^N values observed at any given location, standardized with respect to the centroid of resources in that location by subtracting the resource centroids from the individual isotopic values. All the standardized values were then considered within a single isotopic niche space and isotopic metrics were calculated in order to describe niche metrics and species’ overlap at the meadow scale.

A Bayesian isotopic mixing model available as an open source R package (SIAR, Stable Isotope Analysis in R) was used to assess the relative contributions to consumers’ diets of attached *P. oceanica* leaves, fresh (“Green”) and decomposed (“Brown”) *P. oceanica* leaf litter, epiphytes and sediment organic matter (SOM) (Careddu et al., 2015; Rossi et al., 2015). In accordance with McCutchan, Lewis, Kendall, and McGrath (2003), the isotopic shifts between consumers and resources for δ^13^C and δ^15^N were assumed to be 0.4‰ and 2.3‰, respectively. Metabolic isotopic fractionation by consumers is prohibitively difficult to measure in the field, which would need dedicated feeding experiments (e.g., Madeira, di Lascio, Carlino, Costantini, & Pons, 2013; Rossi, Costantini, & Brilli, 2007). Nevertheless, fractionation has been shown to be predictably influenced by consumer and diet type, consumers’ metabolic pathways, and environmental conditions (Henschke et al., 2015; McCutchan et al., 2003). Thus, considering a set of species relying on a shared pool of potential resources on limited spatial and temporal scales and of similar body size, it can be assumed that differences in specimens’ isotopic signatures reflect differences in their food use (Bašić & Britton, 2016; Fry, 2006; Jackson et al., 2011; Layman, Qattrocchi, et al., 2007; Rossi et al., 2015; Sanders et al., 2015; Swanson et al., 2015; Yao et al., 2016).

At the meadow scale, the proportional contribution of each resource item to the diet of each target species (*P*_*X*meadow_) was calculated as follows:

(1) $$P_{\mathrm{Xmeadow}}=\;\left(P_{XD}*N_{D}+P_{XI}*N_{I}+P_{XF}*N_{F}\right)/\left(N_{D}+N_{I}+N_{F}\right)$$

where *P*_*X*_ is the proportional contribution of resource *X* to the diet; H, I, and L are the sampling locations, and *N* is the population density. This calculation provides a simple weighted measure of the contribution of each resource to the diet of consumers at the meadow scale. It takes account of the spatial (i.e., interlocation) variability of both species density and resource consumption by each species and is not affected by potential isotopic variations in resource items between locations.

Based on diet composition, trophic niche width was measured as the diversity (i.e., Shannon diversity index, Hs) of resources in the diet. Diet similarity between species pairs was calculated by means of the Bray–Curtis similarity index, based on the identity and proportion of resources consumed. As complementary information, the variability among TSs in the consumption of each resource at each location was obtained as the coefficient of variation (C.V.) of the contribution of any given resource to their diet.

### 2.4 Competition strength and carrying capacity

The strength of competition between species pairs was calculated with reference to:

1. The overlap in resource use (Abrams, 1977; Levins, 1968; Pianka, 1969). In this case, competition strength is indicated by β_*ij*_, that is, the effect of species *j* on species *i* (sensu Levins, 1968), with *i* ≠ *j*, in accordance with the formula:
  (2) $$\beta_{ij}=\frac{\sum_{h}pih*pjh}{\sum_{h}{\left(pih\right)}^{2}}$$
  where p*ih* and p*jh* are the proportional consumptions of any given resource *h* by species *i* and species *j*, respectively, obtained as outputs of Bayesian mixing models;
2. The ratio of intraspecific to interspecific isotopic dissimilarity for each species pair, based on mean individual isotopic distances. In this case, competition strength is indicated by α_*ij*_, that is, the effect of species *j* on species *i*, with *i* ≠ *j*, in accordance with the formula:
  (3) $$\alpha_{ij}=\frac{1}{n}\sum_{i=1}^{n}\frac{{\text{ND}}_{ii}}{{\text{ND}}_{ij}}$$
  where ND_*ii*_ is the intraspecific isotopic dissimilarity and ND_*ij*_ the interspecific isotopic dissimilarity of any given specimen of species *i*, and *n* is the number of specimens in *i*. This measure provides a mean value of interaction strength and associated standard error which is dependent on differences in ND_*ii*_ and ND_*ij*_ between specimens. At the species assemblage level (i.e., considering the three TSs and the effect on them of each remaining NTS), the carrying capacity (*K*) for each TS at each sampling location was calculated with reference to Lotka–Volterra competition models:
  (4) $$\frac{dN_{i}}{dt}=r_{i}N_{i}\left(\frac{K_{i}-N_{i}\sum_{i\neq j}^{n}\alpha_{ij}N_{j}}{K_{i}}\right)$$
  and direct competition coefficients coupled with competitors’ densities (Levins, 1968; Pianka, 1974), as follows:
  (5) $$K_{i}=\left(N_{i}+\alpha_{i2}N_{2}+\alpha_{i3}N_{3}+\ldots +\alpha_{ij}N_{j}\right)$$
  where *K*_*i*_ is the carrying capacity for species _*i*_; α_*i*2_, α_*i*3_, …, α_*ij*_ are the effects on species *i* of species 2, 3, …, *j*, respectively, and *N*_*i*_, *N*_2_, *N*_3,_ …, *N*_*j*_ are the densities of species *i*, 2, 3, …, *j,* respectively. In the text, we will refer to the product of α_*ij*_ and *N*_*j*_ as the limiting effect of species *j* on species *i*.

### 2.5 Indirect competition and species assemblage stability

The stability of the species assemblage (i.e., the local Lyapunov stability) was investigated with reference to (1) the community matrix (M), accounting for the pairwise competition coefficients (either β_*ij*_ or α_*ij*_), and (2) the resulting classical Jacobian matrix (J) (May, 1974a; Montoya, Woodward, Emmerson, & Solé, 2009; Whittaker & Levin, 1975). The stability of any given n‐species matrix can be inferred from its eigenvalues, with stability being expected for Jacobian matrices having only negative eigenvalues (in their real part) (Allesina & Tang, 2012; May, 1974b).

In order to account for the effects of both direct and indirect competition, the inverse of the Jacobian matrix (J^−1^) was considered. Each element of J^−1^ describes the net effect of species *j* on species *i*, taking into account all indirect pathways that link species *i* and *j* via intermediate competitors (Montoya et al., 2009; Wootton, 2002). To account for the uncertainty in matrix elements, which is not directly accounted for in inverse Jacobian matrix calculation, we created modified inverse Jacobian matrices to simulate under‐ and overestimates of matrix elements by forcing a substantial (20%) decrease (matrix denoted as ↓J^−1^) or increase (matrix denoted as ↑J^−1^) in each matrix element. In addition, in order to test the effect on species assemblage stability of the distribution of interspecific interaction strengths, a further set of 10 random matrices ($J_{r}^{-1}$) was created by randomly rearranging original off‐diagonal matrix elements. Here, we did not randomize the interspecific interaction strengths (or the isotopic distance and diet composition underlying competition strengths), as this would obviously produce a change in the eigenvalues of the matrix, which would not be informative in this case. On the other hand, randomization of the position of matrix elements made it possible to verify whether alternative stable configurations could exist given the observed magnitude of interaction strengths while ignoring their distribution between species pairs.

## 3 Results

### 3.1 Isotopic distribution, trophic niche, and competition strength

**Figure 1:**
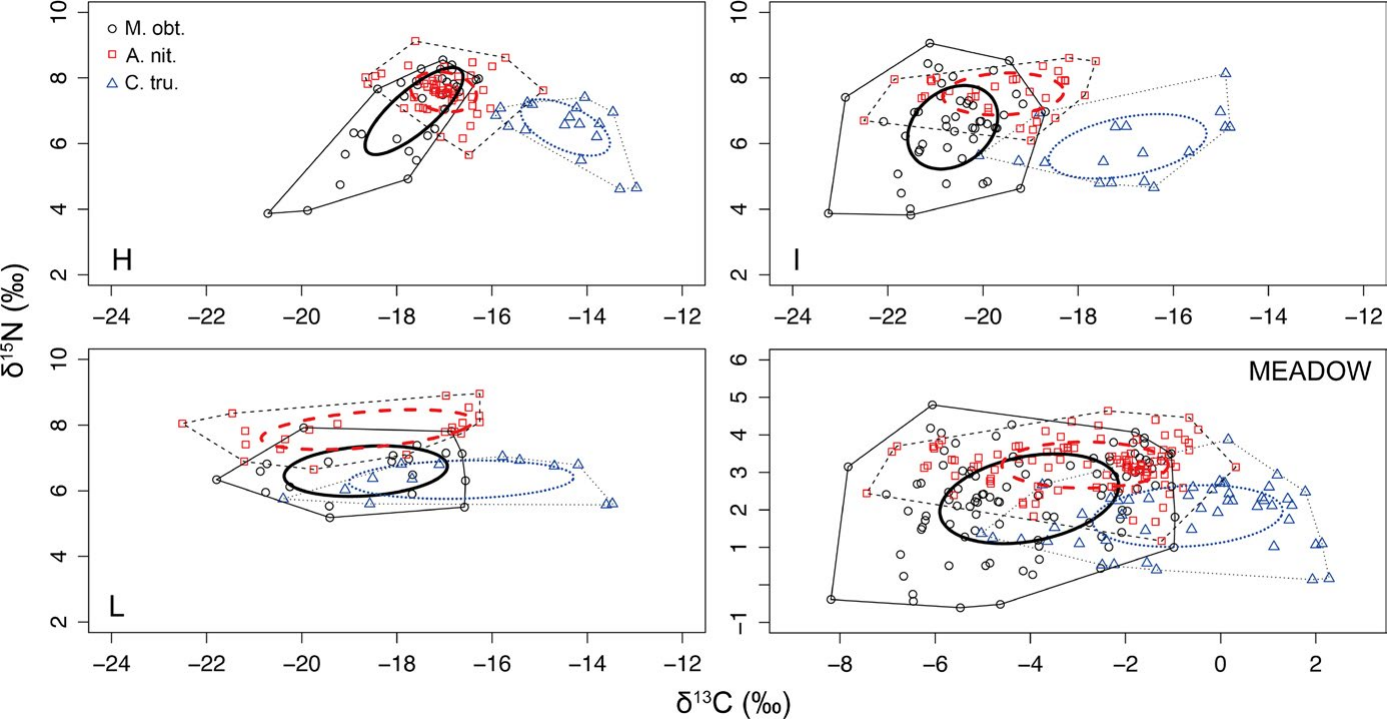
C and N isotopic biplot showing the isotopic distribution of *Microdeutopus obtusatus* (*M. obt*, solid line), *Atanas nitescens* (*A. nit*., dashed line), and *Cimodoce truncata* (*C. tru*, dotted line) in three *Posidonia oceanica* meadow locations differing in coverage (H: high, I: intermediate, and L: low), and at the meadow scale (MEADOW, i.e., when not accounting for spatial segregation of populations between locations). Ellipses represent the isotopic standard ellipse area (SEA) for each population. Polygons represent the isotopic total area (TA) occupied by each population. Values at the meadow scale represent isotopic data standardized with respect to the centroid of the isotopic distribution of resources at each location

Although TS density decreased from H to L, it was not significantly affected by meadow degradation (Kruskal–Wallis test, *M. obtusatus*: Hc = 3.24 *p *=* *.20; *A. nitescens*: Hc = 3.21 *p *=* *.20; *C. truncata*: Hc = 2.12 *p *=* *.35) (Table 1). In contrast, NTS density decreased significantly from H to L (n° of individuals per litterbag: H = 10.7 ± 2.9, I = 4.5 ± 1.0, L = 3.8 ± 0.8; One‐way ANOVA, *F* = 4.35 *p *<* *.05; evenness of nontarget specimens across litterbags in H = 0.81, in I = 0.88, in L = 0.92). Moving from H to L, the isotopic distribution of each TS varied (PERMANOVA, *p *<* *.01) (Figure 1), and both TA and SEAc increased (Table 1). As neither the mean nor the variance (σ^2^) of δ^13^C values differed between sampling sites within each location, for all TSs (Table S2), the values of each TS were pooled between sites and analyzed at the location scale for subsequent comparisons. For all TSs, σ^2^ varied across locations and at the meadow scale (Levene's test for homogeneity of variances, *p* always <.05), whereas the σ^2^ of resources did not vary significantly (Table S3). Specifically, from H to L, σ^2^ increased by 68% for *M. obtusatus*, 89% for *A. nitescens,* and 84% for *C. truncata*, whereas the σ^2^ of resources increased by just 22%, being lowest in I and highest in L. In addition, the variance of the mean δ^13^C of each TS between locations was higher than that of any resource item (Table S3). Similarly, across locations, CR increased by 25% for *M. obtusatus*, 40% for *A. nitescens*, and 57% for *C. truncata*, being lowest in H and highest in L, while the CR of resources increased by just 19%, being lowest in I and highest in L (Table S3). Neither the σ^2^ nor the CR of TSs was affected by the number of isotopic observations (i.e., number of individuals; σ^2^: *R* ^2^ < .01, *p* = .99; CR: *R* ^2^ = .19, *p* = .15).

**Table 1:** Densities and isotopic niche metrics of target species from high‐, intermediate‐, and low‐coverage locations and at the whole‐meadow scale, showing the mean number (±*SE*) of individuals per *Posidonia oceanica* litterbag and evenness of distribution across litterbags

	*Microdeutopus obtusatus*				*Atanas nitescens*				*Cimodoce truncata*			
	H	I	L	Meadow	H	I	L	Meadow	H	I	L	Meadow
No ind./litterbag	10.0 ± 3.1	13.7 ± 5.0	5.3 ± 3.2	9.7 ± 3.1	10.0 ± 2.7	7.2 ± 1.3	4.2 ± 1.2	7.1 ± 1.2	3.8 ± 0.6	2.8 ± 1.3	2.7 ± 0.8	3.1 ± 0.5
Evenness	0.41	0.70	0.65	0.56	0.80	0.91	0.91	0.83	0.94	0.75	0.89	0.86
TA (‰)	8.4	17.8	10.7	30.5	7.2	7.9	9.1	17.4	4.3	10.6	7.0	17.4
SEA (‰)	2.6	3.6	3.9	6.2	1.3	2.5	3.6	3.3	2.0	4.4	4.0	5.1
SEAc (‰)	2.6^a^	3.7^b^	4.1^c^	6.2	1.3^a^	2.5^b^	3.8^c^	3.4	2.1^a^	4.7^b^	4.3^b^	5.2
MND_*ii*_ (‰)	1.90 ± 0.12^a^	1.94 ± 0.01^a^	2.28 ± 0.02^b^	2.76 ± 0.01	1.09 ± 0.01^a^	1.73 ± 0.02^b^	2.68 ± 0.03^c^	2.19 ± 0.01	1.51 ± 0.02^a^	2.34 ± 0.03^b^	2.84 ± 0.05^c^	2.60 ± 0.01
Nontarget α_*ij*_	1.41 ± 0.18^a^	1.19 ± 0.15^a^	1.27 ± 0.12^a^		0.74 ± 0.11^a^	1.18 ± 0.16^b^	1.20 ± 0.15^b^		0.44 ± 0.04^a^	0.74 ± 0.09^b^	0.71 ± 0.15^b^	

TA, total isotopic niche area; SEA, standard ellipse area.

In SEAc, “c” stands for “corrected” by degrees of freedom. MND_*ii*_, mean isotopic distance between conspecific specimens. Nontarget α_*ij*_: mean strength of direct competition with nontarget species. Different superscript letters indicate significant differences (*p *<* *.05; SEAc: Welch's *t*‐test, MND_*ii*_: two‐way ANOVA and Tukey's post hoc comparisons, nontarget α_*ij*_: Kruskal–Wallis test and Mann–Whitney pairwise comparisons).

**Figure 2:**
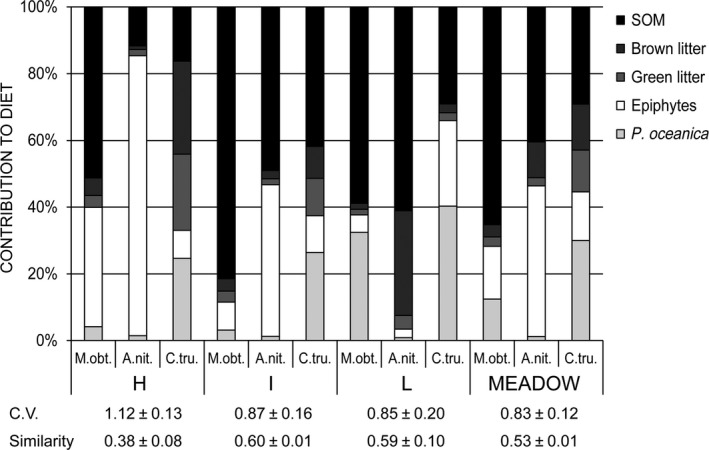
Proportional contribution of each resource to the diet of *Microdeutopus obtusatus* (M. obt), *Athanas nitescen*s (A. nit), and *Cymodoce truncata* (C. tru) in three *P. oceanica* meadow locations differing in coverage (H: high, I: intermediate, and L: low), and at the meadow scale (i.e., when not accounting for spatial segregation of populations between locations). “Brown litter” indicates evidently decomposed *Posidonia oceanica* leaf litter; “Green litter” indicates evidently nondecomposed *P. oceanica* leaf litter. SOM, Sediment Organic Matter. C.V. quantifies the variability in the consumption of each resource by the three target species at each location, whereas “Similarity” is the Bray–Curtis similarity between diets based on the identity and proportion of resources consumed by each species at each location

The MND_*ii*_ differed between TSs, but increased from H to L for all of them (two‐way ANOVA and post hoc comparison, *p *<* *.05), and was not related to their density (*r* ^2^ = .17, *p *=* *.18) (Table 1). At the meadow scale, MND_*ii*_ was higher than that of H and I but was similar to that of L (Kruskal–Wallis and Mann–Whitney comparisons, *p *<* *.05 for all TSs). Resource use varied both between species and between meadow locations (Figure 2). On average, trophic generalism in the use of resources was higher for *C. truncata* (Hs = 1.48 ± 0.10), intermediate for *M. obtusatus* (Hs = 0.90 ± 0.15) and lower in *A. nitescens* (Hs = 0.77 ± 0.13). Mean diet similarity between TS pairs was lowest in H and highest in I and L and at the meadow scale (Figure 2; one‐way ANOVA for repeated‐measures and Tukey's pairwise comparisons, *p* < .05). Accordingly, the variability (i.e., C.V.) in the consumption of each resource among TSs was highest in H and lowest in L and at the meadow scale (Figure 2).

**Figure 3:**
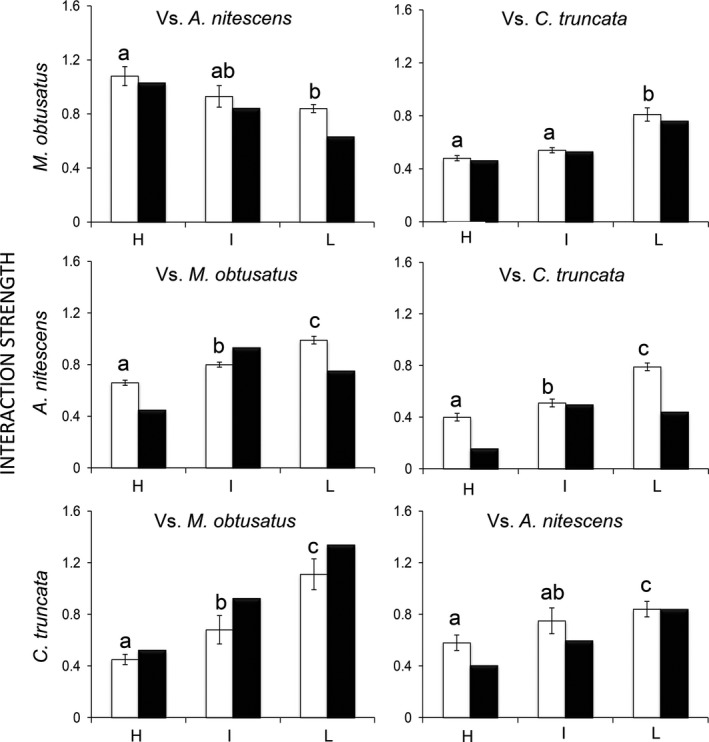
Strength of competition between each species pair at three levels of *Posidonia oceanica* coverage (H: high, I: intermediate, and L: low). Competition strength is measured with reference to (1) overlap in resource use (black bars = β_*ij*_), in accordance with Levins (1968), and (2) intra‐ and interspecific isotopic similarity (white bars = α_*ij*_). Each panel shows the effect of species *j* (at the top) on species *i* (on the left). Different superscript letters indicate significant differences between locations (two‐way ANOVA and post hoc comparisons, *p *<* *.05)

Direct interspecific competition between TSs increased in strength from H to L, the only exception being the effect of *A. nitescens* on *M. obtusatus*, which weakened with falling *P. oceanica* coverage (Figure 3). The values of α_*ij*_ and β_*ij*_ were strongly related (Figure 3), displaying the same pattern of variation in competition strength along the meadow coverage gradient (*r* ^2^ = .88, *p *<* *.0001, slope = 0.91, 95% confidence interval on slope: 0.71, 1.10; *p* slope equal to 1 = 0.41; intercept = 0.12, 95% confidence interval on intercept: −0.02, 0.20). On average (i.e., across locations), *A. nitescens* had the strongest (α_*ij*_ = 0.84 ± 0.10), *M. obtusatus* the intermediate (α_*ij*_ = 0.78 ± 0.14), and *C. truncata* the weakest (α_*ij*_ = 0.59 ± 0.10) competitive effect on the two remaining TSs (Kruskal–Wallis and Mann–Whitney pairwise comparisons, *p *<* *.05). The mean strength of competition with NTSs did not vary with meadow degradation for *M. obtusatus*, whereas it increased significantly from H to L for both *A. nitescens* and *C. truncata* (Table 1). Considering the mean effect of each NTS on the three TSs, the α_*ij*_ values decreased with increasing NTS density (Fig. S2). This pattern was also observed when considering the effect of NTSs on both *A. nitescens* and *M. obtusatus* in isolation, whereas it was less evident for *C. truncata* (Fig. S2).

Competition between *M. obtusatus* (*M*) and *A. nitescens* (*A*) was always asymmetrical, regardless of the degree of meadow coverage, with α_MA_ > α_AM_ in H and I (*t*‐test, *p *<* *.01), and the opposite in L (*t*‐test, *p *<* *.01). In contrast, competition between *C. truncata* and *A. nitescens*, and between *M. obtusatus* and *C. truncata*, was asymmetrical only in H and I, respectively, with *C. truncata* being the subordinate competitor in both cases (*t*‐test, *p *<* *.01). Higher α_*ij*_ values between TS pairs were reflected in higher overlaps between isotopic TAs (*r* ^2^ = .36, *p *<* *.01), but not in higher overlaps between SEAcs (*r* ^2^ = .10, *p* >* *.05) (Table S4). Accordingly, the overlap between TAs was positively correlated with interpopulation diet similarity (*r* ^2^ = .59, *p *<* *.01) and inversely correlated with variability in the consumption of each resource at each location (*r* ^2^ = .97, *p *<* *.01). The α_*ij*_ measured at the meadow scale was significantly higher than the α_*ij*_ measured at the location scale for four of six species–pair interactions (Fig. S3, paired *t*‐test, *p* always <.05). Accordingly, the overlap between TAs was higher at the meadow scale than at the location scale (paired *t*‐test, *t* = 6.4, *p *<* *.01) (Table S4).

### 3.2 Carrying capacity and species assemblage stability

**Figure 4:**
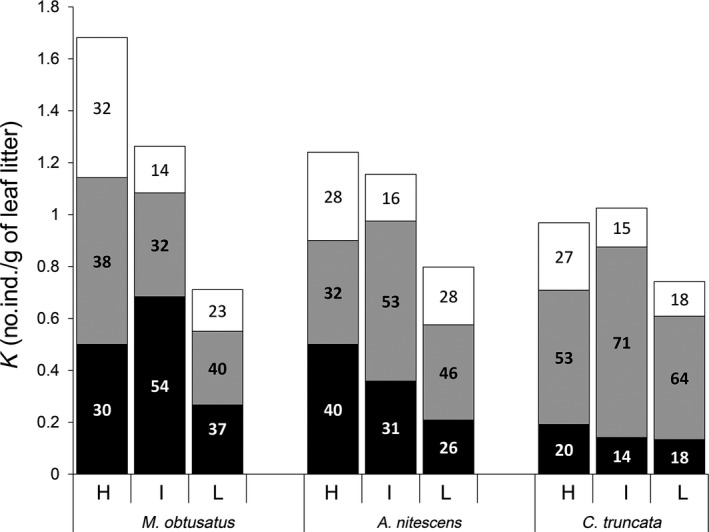
Carrying capacities (*K*) of each target species at three levels of *Posidonia oceanica* coverage. (H: high, I: intermediate, and L: low). For each species, *K* is obtained as the sum of (1) the observed number of specimens (in black), (2) the limiting competitive effect of the remaining target species (in grey), and (3) the limiting competitive effect of the remaining nontarget species (in white). Numbers within each area represent the percentage of the total

Carrying capacity (K) decreased from H to L for all TSs (Figure 4). The mean population density across meadow locations was 40.4 ± 7.2% of K for *M. obtusatus*, 32.5 ± 4.2% for *A. nitescens*, and 17.2 ± 1.8% for *C. truncata*. The limiting effect on each TS of the other two TSs increased with its MND_*ii*_ (*r* ^2^ = .62, *p *<* *.01) and was not related to population density (*r* ^2^ = .004, *p *=* *.87). On the other hand, the limiting effect of NTSs was related neither to TS MND_*ii*_ (*r* ^2^ = .20, *p *=* *.22) nor to TS density (*r* ^2^ = .02, *p *=* *.71), but increased with the evenness of the target specimens’ spatial distribution (i.e., across litterbags) at the location scale (*r* ^2^ = .46, *p *<* *.05). For all possible pairwise interactions between TSs, stable equilibria (as inferred from two‐species competition models, i.e., when *K*_*i*_/α_*ij*_ > *K*_*j*_ and *K*_*j*_/α_*ji*_ > *K*_*i*_) were expected in H and I, but not in L or at the meadow scale (Fig. S4).

The mean competition strength in the community matrix (M) with reference to both β_*ij*_ and α_*ij*_ pairwise competition coefficients was lowest in H and highest in L (Table 2). At the meadow scale, mean competition strength was higher than in H and was similar to competition strength in I (β_*ij*_) and L (α_*ij*_). Considering both β_*ij*_ and α_*ij*_, the leading eigenvalue (λ) of the Jacobian matrix (J) increased from H to L and was >0 at the meadow scale (Table 2). Net competition effects increased species assemblage stability at the location scale (in H, I and L λ^−1^ was always <λ), but not when considering the entire meadow (Table 2). The analysis of both ↓J^−1^ and ↑J^−1^ gave similar results to those obtained with J^−1^ (Table 2). In contrast, the analysis of $J_{r}^{-1}$ failed to replicate the results obtained with J^−1^. Indeed, $\lambda_{r}^{-1}$ was <0 in H ($\lambda_{r}^{-1}$ < 0 for six and five random matrices considering β_*ij*_ and α_*ij*_, respectively) and I ($\lambda_{r}^{-1}$ < 0 for three and four random matrices considering β_*ij*_ and α_*ij*_, respectively), whereas $\lambda_{r}^{-1}$ was always >0 in L (Table 2 and Table S5).

**Table 2:** Mean (±*SE*) interspecific interaction strength between *Microdeutopus obtusatus*,* Atanas nitescens*, and *Cimodoce truncata* in three meadow locations differing in *Posidonia oceanica* coverage (H: high, I: intermediate, L: low), and at the whole‐meadow scale. (A) Values based on isotopic distances between specimens (α_*ij*_). (B) Values based on diet composition obtained as the output of Bayesian mixing models (β_*ij*_). Different superscript letters for $J_{r}^{-1}$ values indicate significant differences (Kruskal–Wallis test and Mann–Whitney pairwise comparisons, (A) Hc = 20.29, *p *<* *.001; (B) Hc = 19.67, *p *<* *.001). “Leading eigenvalue” (L.E.) refers to the real part of the leading eigenvalue of the direct (λ) and inverse (λ^−1^) Jacobian matrix. ↓λ^−1^ and ↑λ^−1^ refer to corrected Jacobian matrices, where a 20% decrease (↓J^−1^) or increase (↑J^−1^) in each original matrix element was applied. $J_{r}^{-1}$ refers to the mean L.E. (±*SE*) of a set of 10 random matrices obtained by re‐arranging original off‐diagonal matrix elements (see Table S5 for details)

(A) Location	α_*ij*_ _mean_	Leading eigenvalue				
		J	J^−1^	↓J^−1^	↑J^−1^	$J_{r}^{-1}$
H	0.61 ± 0.10	−0.09	−1.01	−1.20	−0.81	−0.75 ± 0.59^a^
I	0.69 ± 0.07	−0.06	−0.96	−1.16	−0.77	0.68 ± 0.77^a^
L	0.90 ± 0.05	−0.01	−1.75	−2.10	−1.40	48.72 ± 4.63^b^
Meadow	0.88 ± 0.10	0.03	33.73	40.48	26.98	

(B) Location	β_*ij*_ _mean_	Leading eigenvalue				
		J	J^−1^	↓J^−1^	↑J^−1^	$J_{r}^{-1}$
H	0.50 ± 0.12	−0.06	−27.25	−6.64	−1.52	−0.52 ± 1.90^a^
I	0.71 ± 0.08	−0.08	−5.25	−4.18	−1.12	1.36 ± 1.39^a^
L	0.81 ± 0.12	−0.02	−2.06	−1.65	−2.47	30.52 ± 3.36^b^
Meadow	0.74 ± 0.10	0.21	4.58	3.66	5.50	

## 4 Discussion

Degradation of *P. oceanica* meadow was seen to have a significant effect on the isotopic niche width of macroinvertebrates and diet similarity between species. Both were higher in the low‐coverage meadow patches, with cascade effects on interspecific competition strength and species assemblage stability, despite the same number of total species being found at the intermediate‐ and low‐coverage locations. Habitat loss‐driven increases in trophic dissimilarity between conspecifics imply lower intraspecific competition for food, potentially representing an advantage in conditions of resource shortage and/or stress (Araújo et al., 2008; Bolnick, 2001; Svanbäck & Bolnick, 2007). This suggests that foraging constraints exerted a significant structuring effect on the *P. oceanica* invertebrate community along the habitat degradation gradient. In addition, the declining carrying capacity of the low‐coverage *P. oceanica* patches for the study species supports the hypothesis of limiting conditions associated with meadow degradation (Bell et al., 2001; Boström et al., 2006; Bowden et al., 2001; Calizza, Costantini, et al., 2013; Orth et al., 2006; Van der Heide et al., 2007). It should be noted that in this case, the *K* value obtained for each population represents the lower limit of its potential value, as other kinds of biotic interaction not included in this calculation could further limit the observed population densities. Increased isotopic niche space (TA and SEAc) associated with declining population densities, together with the independence of mean intraspecific isotopic dissimilarity values (MND_*ii*_) from population densities, makes it possible to state confidently that the isotopic niche metrics were not biased by the decreasing number of specimens associated with meadow degradation. Nor does predatory pressure seem to affect our comparison across levels of seagrass coverage. Indeed, while predation may increase in low‐coverage patches due to decreased shelter for prey (Heck & Orth, 2006 and literature cited therein), both trophic niche width and evenness of distribution of specimens across litterbags increased. This is in contrast with the decreased movement of prey and access to resources expected when top‐down control by predators drives prey feeding behavior (Calizza, Rossi, et al., 2013; Lima, 2002). In addition, the absence of differences in the mean and variance of carbon isotopic values of conspecifics between sites within each location suggests no random effects of predation on our results.

Our interpretation of the results is based on the assumption that changes in the value and variance of consumers’ isotopic signatures are mainly dependent on changes in their diet and niche width (Bašić & Britton, 2016; Fry, 2006; Jackson et al., 2011; Layman, Qattrocchi, et al., 2007; Rossi et al., 2015; Sanders et al., 2015; Swanson et al., 2015; Yao et al., 2016). Indeed, isotopic niche width, diet composition, and competition strength were quantified at the location scale, and spatial variation in the isotopic composition of resources between locations was not observed. Furthermore, the isotopic range and variance of resources varied little or not at all between locations, considering both the whole set of resources and each resource item. By contrast, the range and variance of consumer populations’ δ^13^C values increased greatly with *P. oceanica* meadow degradation. In addition, the increase in isotopic niche width across locations differed between target species, further supporting the hypothesis that changes in isotopic niche metrics were a direct consequence of the ecological response of populations to varying *P. oceanica* coverage and associated resource availability. Lastly, the differences in the standardized isotopic distribution of consumers between locations further support the hypothesis that changes in the isotopic signatures reflected spatial compartmentalization of populations and changes in their diet determined by the degree of *P. oceanica* coverage.

It is acknowledged that competition for food is related to specimens’ vagility and ability to explore their foraging home range (Basset, 1995; Corman, Mendel, Voigt, & Garthe, 2015). Taking account of what has been discussed above and the ability of isotopic signatures to provide medium‐ to long‐term information on the resources assimilated by consumers (Fry, 2006), we believe that the temporal and spatial scales underlying our results are appropriate for describing the competitive effects (sensu Levins, 1968) of invertebrate populations on each other. In addition, given that the principal causes of seagrass degradation (trawling, coastal urbanization, and water turbidity due to river runoff) do not affect our sampling area and season, we consider that our results can be safely related to adaptation of the invertebrate populations to long‐term differences in *P. oceanica* coverage.

### 4.1 Stable isotopes and competition strength

The overlap in resource use (β_*ij*_) and the isotopic similarity of populations based on intra‐ and interspecific individual isotopic distances (α_*ij*_) gave equivalent results in terms of the strength and pattern of competitive interactions along the habitat loss gradient. This was also reflected in the direct and indirect effects of competition on species assemblage stability, obtained with direct and inverse Jacobian matrices, respectively. Bayesian mixing models take account of uncertainty in both consumer and resource isotopic signatures, as well as in isotopic fractionation occurring between them. While in the computation of α_*ij*_, it is not possible to account for potential interindividual variability in isotopic fractionation for each population, such potential variability should be considered similar across locations given the spatial scale investigated, and thus does not represent an obstacle to the comparison of populations’ isotopic data across locations (Araújo et al., 2007). In addition, the consistent information obtained by means of Euclidean distances and Bayesian isotopic mixing models can be considered an indirect test of the robustness of our interpretation of results based on isotopic differences between specimens.

α_*ij*_ proposed here and β_*ij*_ as originally proposed by Levins share the same theoretical basis and characteristics. Indeed, (1) α_*ij*_ increases as intraspecific dissimilarity increases with respect to interspecific dissimilarity; (2) MND_*ij*_ and MND_*ji*_ are equivalent, whereas differences in MND_*ii*_ and MND_*jj*_ make α_*ij*_ and α_*ji*_ asymmetric; and (3) α_*ij*_ approaches 1 only when intra‐ and interspecific dissimilarity is equivalent. In turn, (4) α_*ii*_ (i.e., the term on the diagonal in the community matrix) is always 1, regardless of niche width and the number of resources consumed by species *i*. Unlike β_*ij*_, the calculation of α_*ij*_ does not require the isotopic characterization of potential food sources, nor does it require knowledge of the proportional contribution of each food item to the diet of each consumer. Indeed, such information is carried within the isotopic signature of each specimen, which is the result of the relative contribution of all trophic pathways converging in that organism (Bentivoglio et al., 2015; Careddu et al., 2015; Post, 2002; Rossi et al., 2015), thereby explaining the positive isometric correlation between β_*ij*_ and α_*ij*_.

Notably, the use of α_*ij*_ makes it possible to infer competition strength from specimens’ isotopic signatures when: (1) not all potential food sources are known, (2) all potential food sources are known but they are missing from the dataset or cannot be sampled, such as resources obtained by consumers in deep or extreme habitats; (3) medium‐ to long‐term description of the diet based on stomach content analysis or direct observation would be prohibitively difficult, broadening the range of animal groups for which field‐based competition coefficients can be obtained. In addition, calculation of α_*ij*_ makes it possible to quantify both asymmetry in species–pair interactions and the effect of competition on the carrying capacity for each population, based on a measure of isotopic similarity which yields time‐integrated information on foraging behavior at the organism level.

### 4.2 *Posidonia oceanica* habitat loss, niche overlap, and interspecific competition

Considering the three most abundant species in the invertebrate community (i.e., the TSs), the proportional overlap between their isotopic total areas reflected diet similarity and competition strength, which increased with habitat degradation. This was not observed when considering the overlap between standard ellipse areas encompassing the core of the isotopic niche, in accordance with classical niche theory, which does not expect high overlap on the central part of a given resource axis under limiting conditions (Whittaker & Levin, 1975). Asymmetric competition characterized five of nine target species–pair interactions, with *P. oceanica* coverage reduction even inverting the outcome of competition between *A. nitescens* and *M. obtusatus*. Real food webs are expected to exhibit asymmetry in interspecific interactions, potentially stabilizing ecological communities (Rooney et al., 2006). In this case, TS trophic‐functional traits (e.g., intraspecific isotopic similarity), but not demographic ones (e.g., density), were related to differences in competition strength between populations, representing a key aspect linking habitat degradation and species assemblage organization along the disturbance gradient (Boström‐Einarsson et al., 2014; Valiente‐Banuet et al., 2015).

As with competition between TSs, the mean strength of competition between TSs and less abundant (i.e., nontarget) species increased with *P. oceanica* coverage reduction, suggesting that meadow degradation had a widespread effect within the invertebrate community. At the location scale, the limiting effect of NTSs increased with the evenness of the spatial distribution of specimens, which increased with declining *P. oceanica* coverage. This is consistent with the expectation of increased home‐range exploration by consumers in conditions of low‐quality resource patches (Calizza et al., 2012; Pyke et al., 1977). A more even distribution of specimens within their foraging home range as a result of habitat degradation and resource depletion implies a higher probability of species co‐occurrence on each given resource patch. This leads to isotopically close nonconspecifics as a result of increased home range exploration and overlap of resources encountered during harvesting (Basset, 1995; Pyke et al., 1977; Rossi et al., 2015).

### 4.3 Competition, stability, and spatial scale of interactions

Competition between TSs was stronger when measured at the meadow scale than at the location scale, due to increased isotopic and diet similarity when not accounting for the trophic segregation of populations between locations. Spatial patterns and compartmentalization in species interactions have been shown to promote species coexistence, in conjunction with habitat complexity and differences between competitors in home‐range harvesting behavior (Basset, 1995; Durrett & Levin, 1998). At the species assemblage level, stability decreased in the low‐coverage location, due to stronger mean competition and lower carrying capacity. These results imply that (1) habitat degradation lowered the species assemblage's potential to “absorb” environmental changes without destabilization and that (2) although related to *P. oceanica* coverage, stability was low (i.e., λ was negative but close to 0) when considering the direct outcomes of competition alone. At the location scale, the net effect of competition (Basset & Rossi, 1990; Lawlor, 1979) was to increase species assemblage stability, mitigating the negative effect of habitat degradation. Indirect responses mediated by species interactions have the potential to exceed the direct effect of environmental changes at both population and community levels, leading to disturbance propagation or attenuation within the community (Bewick et al., 2014; Montoya et al., 2009). Here, the net effect of species interactions was to reverse the outcomes of competition on population dynamics and stabilize communities (Lawlor, 1979; Montoya et al., 2009). The fact that these results were not affected by potential under‐ or overestimation of matrix elements is indicated by the similar results obtained from the analysis of ↓J^−1^ and ↑J^−1^. On the other hand, the stabilizing inverse relationship between direct and indirect competition became weaker with meadow degradation and was not observed at the meadow scale (see Tables S6 and S7 in the online supplementary material).

Lastly, the random rearrangement of original matrix elements in $J_{r}^{-1}$ provided alternative stable configurations of the species assemblage at high and intermediate *P. oceanica* coverage, but not at low *P. oceanica* coverage, where none of the random configurations satisfied the criteria for stability (i.e., in L, $\lambda_{r}^{-1}$ was always >0). The distribution of interaction strengths between species has been shown to play a major role in community persistence (Montoya et al., 2009; Tang, Pawar, & Allesina, 2014). Our results suggest that under low *P. oceanica* coverage, the species assemblage may have a low probability of recovering a stable configuration following changes in interspecific interactions, such as those potentially associated with species invasion or local extinction. This may have important implications for biodiversity organization and persistence in degraded seagrass meadows, where both habitat degradation and species invasion are expected to increase in the near future (Hemminga & Duarte, 2000; Orth et al., 2006).

## 5 Concluding Remarks

The analyses of C and N isotopic signatures at the individual level and the consideration of intra‐ and interspecific isotopic similarity made it possible to measure competition strength and asymmetry between species pairs in real multispecies communities. This provided information on the effect of seagrass coverage reduction on its carrying capacity with regard to macroinvertebrate populations and the stability of the *P. oceanica* species assemblage, improving our understanding of mechanisms that contribute to biodiversity decline following habitat degradation. Indeed, the functional response of populations along the disturbance gradient promoted interspecific competition, eroding community stability. Under these conditions, the inclusion of additional species within a community of generalist populations with highly overlapping niches is unlikely to be stable and therefore is not expected to be observed (Borrelli et al., 2015; May, 1974a).

The number of stable isotope‐based ecological studies is rapidly increasing, addressing a huge range of habitats and taxonomic groups (Fry, 2006; Mancinelli & Vizzini, 2015; Rossi et al., 2015). We demonstrated that the isotopic approach could be useful in order to describe changes in trophic interactions and community dynamics along disturbance gradients. These calculations are not limited to two‐dimensional isotopic niches and Euclidean distances, but are extendible to any given set of ecological niche axes for which specimens’ positions and pairwise distances between potentially competing organisms can be measured. Information on the strength of competition between populations and stability at the community level will provide insights regarding the link between variations in the organization of communities and the effect of disturbance on habitat carrying capacity, as well as on the persistence mechanisms of biodiversity in the face of environmental changes.

## Conflict of Interest

None declared.

## Author Contributions

E.C., M.L.C., and L.R. conceived the study. E.C., G.C., and L.R. performed the sampling. E.C. and G.C. performed laboratory and statistical analyses. E.C., M.L.C., and L.R wrote and revised the manuscript.
