# Supplementary material for: Effect of habitat degradation on competition, carrying capacity, and species assemblage stability
Source: Ecol Evol. 2017 Jun 17;7(15):5784–96. doi: 10.1002/ece3.2977 (PMC5552933; doi:10.1002/ece3.2977)
Supplement: Supplementary file 1 [file ECE3-7-5784-s001.docx]

**SUPPLEMENTARY MATERIAL**

**Effect of habitat degradation on competition, carrying capacity and species assemblage stability**

Edoardo Calizza^1,2*^, Maria Letizia Costantini^1,2^, Giulio Careddu^1^, Loreto Rossi^1,2^

^1^Department of Environmental Biology, Sapienza University of Rome, Via dei Sardi 70, Rome (IT)

^2^CoNISMa, Piazzale Flaminio 9, Rome (IT)

*Corresponding Author: [edoardo.calizza@uniroma1.it](mailto:edoardo.calizza@uniroma1.it)

**Table S1** Modified with permission from Calizza et al. (2013a). Isotopic signatures (‰) of basal resources across three meadow locations differing in *P. oceanica* coverage (H: high coverage; 92.5±2.5%; I: intermediate coverage, 70.0±5.0%; L: low coverage, 50.0±5.0%). “Green” refers to fresh, evidently not decomposed *P. oceanica* leaf litter; “Brown” refers to brown, evidently decomposed *P. oceanica* leaf litter. S.O.M.: sediment organic matter.

|  |  |  |  | **H** | **I** | **L** |
| --- | --- | --- | --- | --- | --- | --- |
| ***P. oceanica* leaves** | | δ^13^C |  | -12.3±0.3 | -12.1±0.4 | -12.7±0.2 |
|  |  | δ^15^N |  | 4.2±0.2 | 3.1±0.3 | 3.0±0.3 |
| **Epiphytes** | | δ^13^C |  | -17.2±0.1 | -17.0±0.1 | -16.8±0.3 |
|  |  | δ^15^N |  | 5.5±0.1 | 5.3±0.4 | 4.5±0.3 |
| **"Green" leaf litter** | | δ^13^C |  | -11.7±0.4 | -11.6±0.4 | -11.1±0.3 |
|  |  | δ^15^N |  | 4.6±0.2 | 4.2±0.3 | 4.8±0.4 |
| **"Brown" leaf litter** | | δ^13^C |  | -12.5±0.4 | -12.6±0.2 | -13.4±0.2 |
|  |  | δ^15^N |  | 3.3±0.3 | 4.7±0.3 | 5.0±0.2 |
| **S.O.M.** |  | δ^13^C |  | -22.5±0.3 | -22.0±0.4 | -24.0±0.4 |
|  |  | δ^15^N |  | 4.8±0.3 | 4.0±0.4 | 5.2±0.5 |

**Table S2** a: Effect of sampling location and site on mean δ^13^C value of each target species. M. obt.: *Microdeutopus obtusatus*; A. nit.: *Athanas nitescens*; C. tru.: *Cymodoce truncata*. Three sampling locations differing in their degree of *P. oceanica* coverage and two sampling sites within each location where considered. For details please refer to the materials and methods section in the main text. b: F test on variances of δ^13^C values of each target species between sampling sites within each sampling location (H, I, L). For comparison of isotopic variances between locations and species please refer to the results section in the main text and to Table S3. Bold values indicate a significant effect, for p < 0.05.

| a |  |  |  |  |  |  |  | b |  |  |  |
| --- | --- | --- | --- | --- | --- | --- | --- | --- | --- | --- | --- |
| Species | Factor | Sumsqrs | df | Meansqr | F | p value |  | Species | Location | F | p value |
| M. obt. | Location | 183.50 | 2 | 91.73 | 68.92 | **3.75E-19** |  | M. obt. | H | 1.41 | 0.45 |
|  | Site | 0.76 | 1 | 0.76 | 0.57 | 0.45 |  |  | I | 1 | 0.99 |
|  | Interaction | 0.83 | 2 | 0.42 | 0.31 | 0.73 |  |  | L | 1.3 | 0.67 |
| A. nit. | Location | 137.70 | 2 | 68.84 | 42.66 | **4.01E-14** |  | A. nit. | H | 1.55 | 0.28 |
|  | Site | 1.14 | 1 | 1.143 | 0.71 | 0.40 |  |  | I | 1.6 | 0.37 |
|  | Interaction | 4.67 | 2 | 2.33 | 1.45 | 0.24 |  |  | L | 1.12 | 0.86 |
| C. tru. | Location | 61.60 | 2 | 30.80 | 11.37 | **1.18E-04** |  | C. tru. | H | 1.64 | 0.5 |
|  | Site | 2.67 | 1 | 2.67 | 0.99 | 0.33 |  |  | I | 2.25 | 0.25 |
|  | Interaction | 1.72 | 2 | 0.86 | 0.32 | 0.73 |  |  | L | 1.23 | 0.84 |

**Table S3** Range (i.e. difference between maximum and minimum values, CR) and variance (σ^2^) of δ^13^C signatures of target species and resources at each sampling location (H, I, L) and at the whole-meadow scale (MEADOW). Δ% indicates the % increase in σ^2^ and CR based on the lowest and highest values observed. The variance across locations is calculated from the mean value of each item at each location. Different upper case letters indicate a significant difference in σ^2^ between locations (Levene’s test for homogeneity of variances and F-test pairwise comparisons, p< 0.05)

|  |  | H | I | L | MEADOW | Δ% | σ^2^ across locations |
| --- | --- | --- | --- | --- | --- | --- | --- |
| *M. obtusatus* | σ^2^ | 1.0^a^ | 1.0^a^ | 2.8^b^ | 3.5^b^ | 67.9 | 2.3 |
|  | CR | 5.3 | 4.8 | 6.4 | 7.2 | 25.2 |  |
| *A. nitescens* | σ^2^ | 0.5^a^ | 1.6^b^ | 4.7^c^ | 3.0^c^ | 89.4 | 1.7 |
|  | CR | 3.7 | 4.9 | 6.3 | 7.8 | 40.3 |  |
| *C. truncata* | σ^2^ | 0.8^a^ | 2.6^b^ | 5.1^c^ | 3.9^bc^ | 84.3 | 1.9 |
|  | CR | 3.0 | 5.3 | 6.9 | 7.3 | 57.3 |  |
| Resources | σ^2^ | 21.3^a^ | 19.7^a^ | 25.3^a^ | 22.2^a^ | 22.1 | 0.1 |
|  | CR | 10.8 | 10.4 | 12.9 | 12.9 | 19.4 |  |
| *P. oceanica* |  |  |  |  |  |  | 0.1 |
| Epiphytes |  |  |  |  |  |  | 0.0 |
| Green_litter |  |  |  |  |  |  | 0.1 |
| Brown_litter |  |  |  |  |  |  | 0.2 |
| SOM |  |  |  |  |  |  | 1.0 |

**Table S4** Overlap in isotopic total area (TA) and standard ellipse area (SEAc, where “c” stands for “corrected” for degrees of freedom) (SIBER analysis, Jackson et al., 2011) for each species pair in three meadow locations differing in *P. oceanica* coverage (H: high, I: intermediate, L: low). As an example, “M vs. A” indicates the overlap between *M. obtusatus* (M) and *A. nitescens* (A) with respect to the niche area of *M. obtusatus*.

| **OVERLAP (%)** |  | **TA** | **SEAc** | **TA** | **SEAc** |
| --- | --- | --- | --- | --- | --- |
|  |  | vs. A | | vs. C | |
| *M. obtusatus* (M) | H | 29.5 | 33.7 | 0.0 | 0.0 |
|  | I | 33.2 | 20.5 | 5.5 | 0.0 |
|  | L | 32.0 | 0.5 | 30.3 | 32.9 |
|  | Meadow | 46.1 | 27.4 | 25.3 | 0.6 |
|  |  | vs. M | | vs. C | |
| *A. nitescens* (A) | H | 34.5 | 68.5 | 2.6 | 0.0 |
|  | I | 74.3 | 29.5 | 6.4 | 0.0 |
|  | L | 37.5 | 0.5 | 0.0 | 0.0 |
|  | Meadow | 80.6 | 51.0 | 38.3 | 0.0 |
|  |  | vs. M | | vs. A | |
| *C. truncata* (C) | H | 0.0 | 0.0 | 4.5 | 0.0 |
|  | I | 9.3 | 0.0 | 4.8 | 0.0 |
|  | L | 46.4 | 31.1 | 0.0 | 0.0 |
|  | Meadow | 44.3 | 0.8 | 38.5 | 0.0 |

**Table S5** Leading eigenvalues of random inverse Jacobian matrices (J_r_^-1^) obtained by randomly re-arranging off-diagonal elements of the original Jacobian matrix at each sampling location (H, I and L). H: high *P. oceanica* meadow coverage (92.5±2.5%); I: intermediate meadow coverage (70.0±5.0%); L: low meadow coverage (50.0±5.0%). a: matrices obtained from α_ij_ values of interaction strength; b: matrices obtained from β_ij_ values of interaction strength. Leading eigenvalues of original inverse Jacobian matrices (J^-1^) are shown for comparison (see Table 3 in the main text). Bold values indicate matrices where stability is achieved (i.e. local Lyapunov stability, when the leading eigenvalue < 0).

| a | **Leading Eigenvalue** | | |  | b | **Leading Eigenvalue** | | |
| --- | --- | --- | --- | --- | --- | --- | --- | --- |
| **J_r_^-1^** | **H** | **I** | **L** |  | **J_r_^-1^** | **H** | **I** | **L** |
| 1 | **-2.66** | **-2.52** | 27.79 |  | 1 | **-7.76** | **-5.73** | 14.10 |
| 2 | **-2.59** | **-2.12** | 33.08 |  | 2 | **-7.39** | **-4.9** | 15.48 |
| 3 | **-2.40** | **-1.17** | 36.33 |  | 3 | **-4.00** | **-2.06** | 26.90 |
| 4 | **-2.32** | **-1.06** | 39.41 |  | 4 | **-3.82** | 0.25 | 27.30 |
| 5 | **-2.27** | 0.01 | 46.54 |  | 5 | **-3.13** | 1.69 | 29.17 |
| 6 | 0.21 | 0.74 | 47.66 |  | 6 | **-1.00** | 3.27 | 31.09 |
| 7 | 0.47 | 2.36 | 59.37 |  | 7 | 0.11 | 3.37 | 33.94 |
| 8 | 1.26 | 2.38 | 61.59 |  | 8 | 3.46 | 5.28 | 37.04 |
| 9 | 1.37 | 4.05 | 66.55 |  | 9 | 8.48 | 5.39 | 42.47 |
| 10 | 1.40 | 4.09 | 68.84 |  | 10 | 9.88 | 6.99 | 47.72 |
| Mean | **-0.75** | 0.68 | 48.72 |  | mean | **-0.52** | 1.36 | 30.52 |
| **J^-1^** | **-1.01** | **-0.96** | **-1.95** |  | **J^-1^** | **-27.25** | **-5.25** | **-2.06** |

**Table S6** Linear regressions between the elements of the direct Jacobian matrix (x) and corresponding elements in the inverse Jacobian matrix (y) in three *P. oceanica* meadow Locations (H, I, L), and at the whole-meadow scale (Meadow). H: high meadow coverage (92.5±2.5%); I: intermediate meadow coverage (70.0±5.0%); L: low meadow coverage (50.0±5.0%). n.s. = not significant, when *p* > 0.1. a: matrices obtained from α_ij_ values of interaction strength; b: matrices obtained from β_ij_ values of interaction strength.

| *a* | **Model** | **r^2^** | ***p* value** |
| --- | --- | --- | --- |
| **H** | y = -13.03x-0.78 | 0.95 | 0.0009 |
| **I** | y = -13.16x+0.16 | 0.57 | 0.07 |
| **L** | n.s. | 0.36 | 0.21 |
| **Meadow** | y = 59.55x+11.10 | 0.67 | 0.04 |
| *b* |  |  |  |
| **H** | y = -40.79x-3.39 | 0.69 | 0.03 |
| **I** | y = -13.61x-1.58 | 0.89 | 0.004 |
| **L** | n.s. | 0.52 | 0.11 |
| **Meadow** | n.s. | 0.23 | 0.32 |

These results, combined with the sign structure of the inverse Jacobian matrices (Table S4), imply that the degree of species assemblage organisation (sensu Lawlor 1979) was reduced by meadow degradation. In H, all off-diagonal elements of J^-1^ varied in sign with respect to the corresponding values in J, with a significant inverse relationship between direct and inverse matrix elements, whereas six elements in I and four elements in L varied in sign, with non-significant relationships observed in L. At the meadow scale, only two out of six off-diagonal elements of J^-1^ varied in sign, with J and J^-1^ elements being unrelated (in b) or even positively related (in a), implying a lack of organisation and high instability of the species assemblage at this wider spatial scale.

**Table S7** Sign structure of the inverse Jacobian matrices (J^-1^) describing the net effect of interspecific competition between the three most abundant species in three *P. oceanica* meadow locations (H, I, L), and at the whole-meadow scale (Meadow). H: high meadow coverage (92.5±2.5%); I: intermediate meadow coverage (70.0±5.0%); L: low meadow coverage (50.0±5.0%). Direct competition is assumed to be described by a negative sign. Therefore, a positive sign between a species pair indicates that the indirect effects of competition invert the direct ones, with a net positive effect being observed (Montoya et al., 2009).

| **H** | *M. obtusatus* | *A. nitescens* | *C. truncata* | **I** | *M. obtusatus* | *A. nitescens* | *C. truncata* |
| --- | --- | --- | --- | --- | --- | --- | --- |
| *M. obtusatus* |  | **+** | **+** | *M. obtusatus* |  | **+** | **+** |
| *A. nitescens* | **+** |  | **+** | *A. nitescens* | **+** |  | **+** |
| *C. truncata* | **+** | **+** |  | *C. truncata* | **+** | **+** |  |
| **L** | *M. obtusatus* | *A. nitescens* | *C. truncata* | **Meadow** | *M. obtusatus* | *A. nitescens* | *C. truncata* |
| *M. obtusatus* |  | **+** | **+** | *M. obtusatus* |  | **-** | **-** |
| *A. nitescens* | **+** |  | **-** | *A. nitescens* | **-** |  | **+** |
| *C. truncata* | **+** | **-** |  | *C. truncata* | **-** | **+** |  |

**Fig. S1** Direct competition strength (α_ij_) between each non-target species and *M. obtusatus*, *A. nitescens* and *C. truncata*. Each symbol represents the effect of one non-target species expressed in relation to its density. “Mean effect” in the upper-left panel refers to the mean effect of each non-target species on the three target species. Regression models are shown when significant (*p*< 0.05). Please note differences on the y-axis between panels.

**Fig. S2** Comparison of mean (±s.e.) interaction strength (α_ij_, i.e. the effect of species *j* on species *i*) at the location and meadow scales. Stars indicate significant differences between spatial scales (paired t-test, *: *p*< 0.05, **: *p*< 0.01, ***: *p*< 0.001). A. ni.: *Athanas nitescens*. C. tr.: *Cymodoce truncata*. M. ob*.: Microdeutopus obtusatus.*

**Fig. S3** Zero-growth isoclines derived from classic Lotka-Volterra two-species competition models (software: Populus 5.5, College of Biological Sciences, University of Minnesota, Minneapolis, MN) at different degrees of *P. oceanica* coverage (H: high, I: intermediate, and L: low) and at the whole-meadow scale. M: *M. obtusatus*; A: *Atanas nitescens*; C: *C. truncata*. A stable equilibrium is obtained when isoclines cross and Ki/αij > Kj and Kj/αji > Ki. Empty green symbols are the measured population densities in the field, whereas full green symbols are expected densities at equilibrium, considering direct competition between species pairs. Green question marks indicate that the outcome of competition could not be unambiguously predicted and is dependent on population densities. Expected densities at equilibrium are shown purely to allow better visualisation of the theoretical outcome of each species-pair interaction, and are not intended to represent the values one would expect to observe in the field when in the presence of other competitors.
